# Trends in pancreatic cancer mortality in the United States 1999–2020: a CDC database population-based study

**DOI:** 10.1007/s10552-024-01906-z

**Published:** 2024-08-19

**Authors:** Alexander J. Didier, Swamroop Nandwani, Alan M. Fahoury, Daniel J. Craig, Dean Watkins, Andrew Campbell, Caleb T. Spencer, Macelyn Batten, Divya Vijendra, Jeffrey M. Sutton

**Affiliations:** 1 The University of Toledo College of Medicine and Life Sciences, 3000 Arlington Ave, Toledo, OH 43614 USA; 2 Division of Oncologic and Endocrine Surgery, Department of Surgery, Medical University of South Carolina, Charleston, SC USA; 3 Division of Hematology & Oncology, Department of Medicine, The University of Toledo College of Medicine and Life Sciences, Toledo, OH USA

**Keywords:** Pancreatic cancer, Mortality, Disparities, Rural populations, Urban populations, Sociodemographic factors

## Abstract

**Introduction:**

Pancreatic cancer is a significant public health concern and a leading cause of cancer-related deaths worldwide. This study aimed to investigate pancreatic cancer mortality trends and disparities in the United States (US) from 1999 to 2020.

**Methods:**

Data were obtained from the Centers for Disease Control (CDC) Wide-Ranging Online Data for Epidemiologic Research database. Mortality rates were age-adjusted and standardized to the year 2000 US population. Joinpoint regression was used to analyze temporal trends in age-adjusted mortality rates (AAMRs) by sociodemographic and geographic variables.

**Results:**

Between 1999 and 2020, pancreatic cancer led to a total of 810,628 deaths in the US, an average mortality of nearly 39,000 deaths per year. The AAMR slightly increased from 10.6 in 1999 to 11.1 in 2020, with an associated annual percent change (APC) of 0.2. Mortality rates were highest among individuals aged 65 and older. Black individuals experienced the highest overall pancreatic cancer-related AAMR at 13.8. Despite this, Black individuals experienced a decreasing mortality trend over time (APC −0.2) while White individuals experienced an increasing trend in mortality (APC 0.4). Additionally, individuals residing in rural areas experienced steeper rates of mortality increase than those living in urban areas (APC 0.6 for rural vs −0.2 for urban). White individuals in urban and rural populations experienced an increase in mortality, while Black individuals in urban environments experienced a decrease in mortality, and Black individuals in rural environments experienced stable mortality trends.

**Conclusions:**

Mortality from pancreatic cancer continues to increase in the US, with racial and regional disparities identified in minorities and rural-dwelling individuals. These disparate findings highlight the importance of ongoing efforts to understand and address pancreatic cancer treatment and outcomes disparities in the US, and future studies should further investigate the underlying etiologies of these disparities and potential for novel therapies to reduce the mortality.

## Introduction

Pancreatic cancer is one of the most lethal malignancies, with dismal 5-year survival rates of 11.5% [1]. Despite only accounting for 3.2% of all new cancer cases, pancreatic cancer—the vast majority (90%) of which are diagnosed as pancreatic ductal adenocarcinoma—led to 8.2% of all cancer deaths in 2022 [1, 2]. This is largely due to the fact that pancreatic cancer often presents at an advanced stage, rendering itself surgically unresectable and restricting treatment options to systemic therapies with limited long-term efficacy.

Despite aggressive surgical efforts coupled with systemic therapies and a new frontier of modern targeted therapies, pancreatic cancer survival trends have remained relatively stagnant [1, 3]. In the context of an increasing global and national incidence of pancreatic cancer, a nuanced understanding of trends in mortality may allow researchers and clinicians to recognize drivers of mortality, especially when considering population-level sociodemographic differences [4]. Previous studies have shown worsened survival among vulnerable populations, with non-Hispanic Black patients experiencing 20% worse survival compared with non-Hispanic White patients [5]. While incidence and survival data are known, demographic and geographic trends in pancreatic cancer mortality are largely understudied. The investigation of population level sociodemographic differences may inform both clinicians and epidemiologists in the development of state-level health policy developments towards pancreatic cancer equity in the United States. Additionally, there is a paucity of granular data in the literature, with few studies exploring the intersection between sociodemographic and geographic variables. In the current study, we utilized a nationwide database linked to death certificates to describe the recent trends in pancreatic cancer mortality in the United States (US) population between 1999 and 2020 to provide an updated analysis of emerging population trends.

## Materials and methods

### Data collection

The US Centers for Disease Control (CDC) Wide-Ranging Online Data for Epidemiologic Research (WONDER) database is a publicly available dataset which collects mortality etiology data from death certificates and has been previously validated in studies investigating mortality related to cancer [6–9]. For the current study, the CDC WONDER dataset was queried to identify incidences of mortality attributed to pancreatic cancer (International Classification of Diseases, 10th edition [ICD-10] code C25.x) between the years 1999–2020.

These data were further stratified based on demographic variables including age (25–44, 44–65, > 65), sex (male or female), and race (White, Black, Hispanic, Asian or Pacific Islander, or Native American/American Indian). Sex and race data were collected from death certificates. In addition, regional variables including geographic density (urban [population ≥ 1 million], suburban [population 50,000–999,999], and rural [population < 50,000]), as well as geographic region (Northeast, Midwest, South, and West) [10] were queried to assess for regional disparities in pancreatic-cancer related mortality.

### Age-adjusted mortality rate statistical analysis

Pancreatic cancer age-adjusted mortality rates (AAMRs) (i.e., number of deaths per 100,000 people) were calculated from data extracted from the dataset and were standardized to the year 2000 US population [11]. Mortality rate was controlled for across age groups (i.e., 25–44, 45–64, 65 + years) to adjust for the influence that different age distributions may have on death rates. We then performed subgroup analysis based on the previous mentioned demographic variables. Additional sensitivity analysis was performed to assess the intersectionality of race and rurality in Non-Hispanic White and Non-Hispanic Black populations in rural and urban areas. Other races were excluded from sensitivity analysis due to small sample size in the rural subgroup. Joinpoint Regression software (National Cancer Institute, Bethesda, MD) was then used to identify any temporal trends in the AAMR over the study time period [12]. Joinpoint regression identifies significant changes in the AAMR over time through a Monte Carlo permutation method to identify the optimal number of line segments connecting two data points [13]. Following this, the software segments the entire time period by those joinpoints and estimates an annual percent change (APC) for each segment. The APC and average annual percent change (AAPC) were calculated, with an APC of 1.0 corresponding to a 1.0% annual increase and an APC of −1.0 corresponding with an annual 1.0% decrease in mortality for a given year. APCs were considered significantly increasing or significantly decreasing if the slope describing the change in mortality was significantly different than 0 using 2‐tailed t-testing. Statistical significance was set at *p* < 0.05.

This study did not require institutional review board approval because the CDC WONDER is a publicly available database that contains de-identified data. The data that support the findings of this study are openly available at https://wonder.cdc.gov.

## Results

Between 1999 and 2020, pancreatic cancer led to 810,628 deaths in the United States, an average annual mortality of nearly 39,000 deaths per year. In 1999, the pancreatic cancer AAMR was 10.6 per 100,000, rising to an AAMR of 11.1 in 2020, yielding an associated APC of 0.2 (95% CI 0.2 to 0.3).

**Fig. 1:**
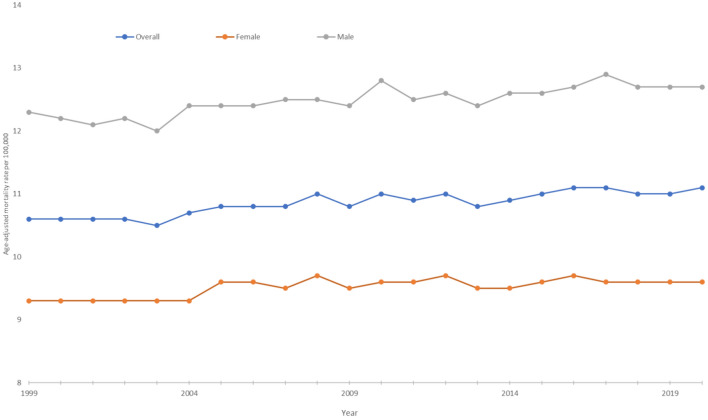
Trends in age‐adjusted, pancreatic cancer‐related mortality rates stratified by sex in the United States, 1999–2020. Overall: 1999–2020 APC 0.24* (95% CI 0.2–0.3); Female: 1999–2008 APC 0.43* (95% CI 0.2–0.7), 2008–2020 APC 0.01 (95% CI −0.1 to 0.2); Male: 1999–2020 APC 0.2* (95% CI 0.2–0.3). *Indicates that the annual percentage change (APC) is significantly different from 0 at *α* = 0.05

Between 1999 and 2020, 409,123 men and 401,505 women died due to pancreatic cancer. During this time period, the average AAMR was 12.5 for men and 9.5 for women. The AAMR rose from 1999 to 2020 in both groups, climbing from 9.3 to 9.6 in women with an associated APC of 0.2 (95% CI 0.1–0.2) and rising from 12.3 to 12.7 in men with an APC of 0.2 (95% CI 0.2–0.3) (Fig. 1).

**Fig. 2:**
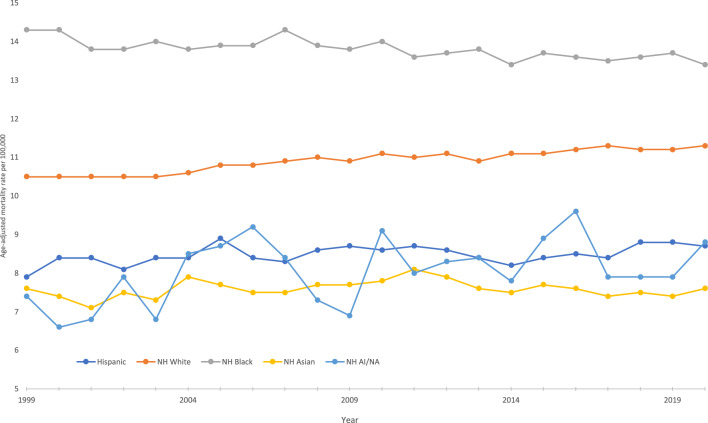
Trends in age‐adjusted, pancreatic cancer‐related mortality rates stratified by race in the United States, 1999–2020. Hispanic: 1999–2020 APC 0.21* (95% CI 0.0–0.4); NH White 1999–2008 APC 0.6* (95% CI 0.3–0.8), 2008–2020 APC 0.3* (95% CI 0.1–0.4); NH Black 1999–2020 APC −0.2* (95% CI to −0.3 to −0.1); NH Asian 1999–2011 APC 0.5* (95% CI 0.0–1.0), 2011–2020 APC −0.6* (95% CI −1.0 to −0.1); NH AI/AN 1999–2020 APC 0.6 (95% CI −0.0 to 1.3). *Indicates that the annual percentage change (APC) is significantly different from 0 at *α* = 0.05

Between 1999 and 2020, Non-Hispanic Black individuals experienced the highest pancreatic cancer-related AAMR at 13.8 deaths per 100,000, followed by Non-Hispanic White individuals at 10.9. The AAMR for the Non-Hispanic Black cohort decreased from 14.3 in 1999 to 13.4 in 2020 with an associated APC of −0.2 (95% CI −0.3 to −0.1), while the AAMR rose or stayed the same for all other groups. In the Non-Hispanic White cohort, the AAMR rose from 10.5 in 1999 to 11.3 in 2020 with an APC of 0.4 (95% CI 0.3–0.4). The AAMR for the Hispanic cohort rose from 7.9 in 1999 to 8.7 in 2020 with an APC of 0.2 (95% CI 0.0–0.4), while the AAMR for the Non-Hispanic American Indian/Alaskan Native cohort rose from 7.4 to 8.8 with an APC of 0.6 (95% CI −0.1 to 1.3). In the Non-Hispanic Asian/Pacific Islander population, the AAMR remained steady at 7.6 throughout the study period, with an APC of 0.1 (95% CI −0.2 to 0.2) (Fig. 2).

**Fig. 3:**
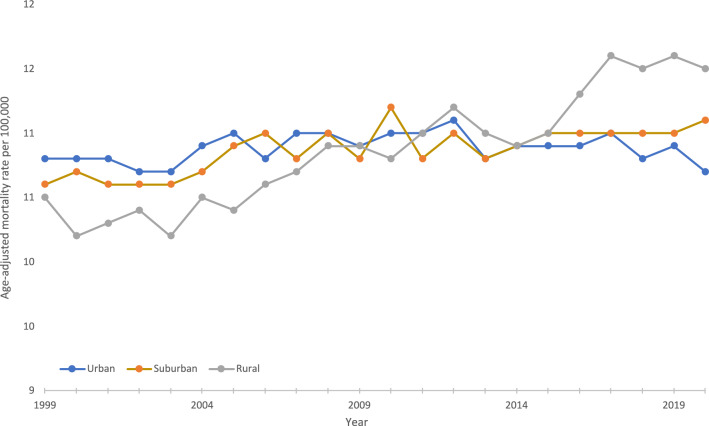
Trends in age‐adjusted, pancreatic cancer‐related mortality rates stratified by geographic density in the United States, 1999–2020. Urban: 1999–2011 APC 0.2* (95% CI 0.0–0.4), 2011–2020 APC −0.2 (−0.5 to 0.0); Suburban: 1999–2020 APC 0.2* (95% CI 0.1–0.3); Rural: 1999–2020 APC 0.6* (95% CI 0.5–0.7). *Indicates that the annual percentage change (APC) is significantly different from 0 at *α* = 0.05

During the study period, the overall AAMR for urban, suburban, and rural subgroups was 10.9 deaths per 100,000 attributed to pancreatic cancer. The AAMR for the urban subgroup slowly rose from 10.8 in 1999 to 11.0 in 2011 with an APC of 0.2 (95% CI 0.1–0.3), then slightly fell to 10.7 in 2020 with an associated APC of −0.2 (95% CI −0.5 to 0.0). For the suburban subgroup, the AAMR rose from 10.6 in 1999 to 11.1 in 2020 with an APC of 0.2 (95% CI 0.1–0.3). The AAMR for the rural subgroup rose from 10.5 in 1999 to 11.5 in 2020 with an APC of 0.6 (95% CI 0.5–0.7), the highest APC of any geographic density subgroup (Fig. 3).

**Fig. 4:**
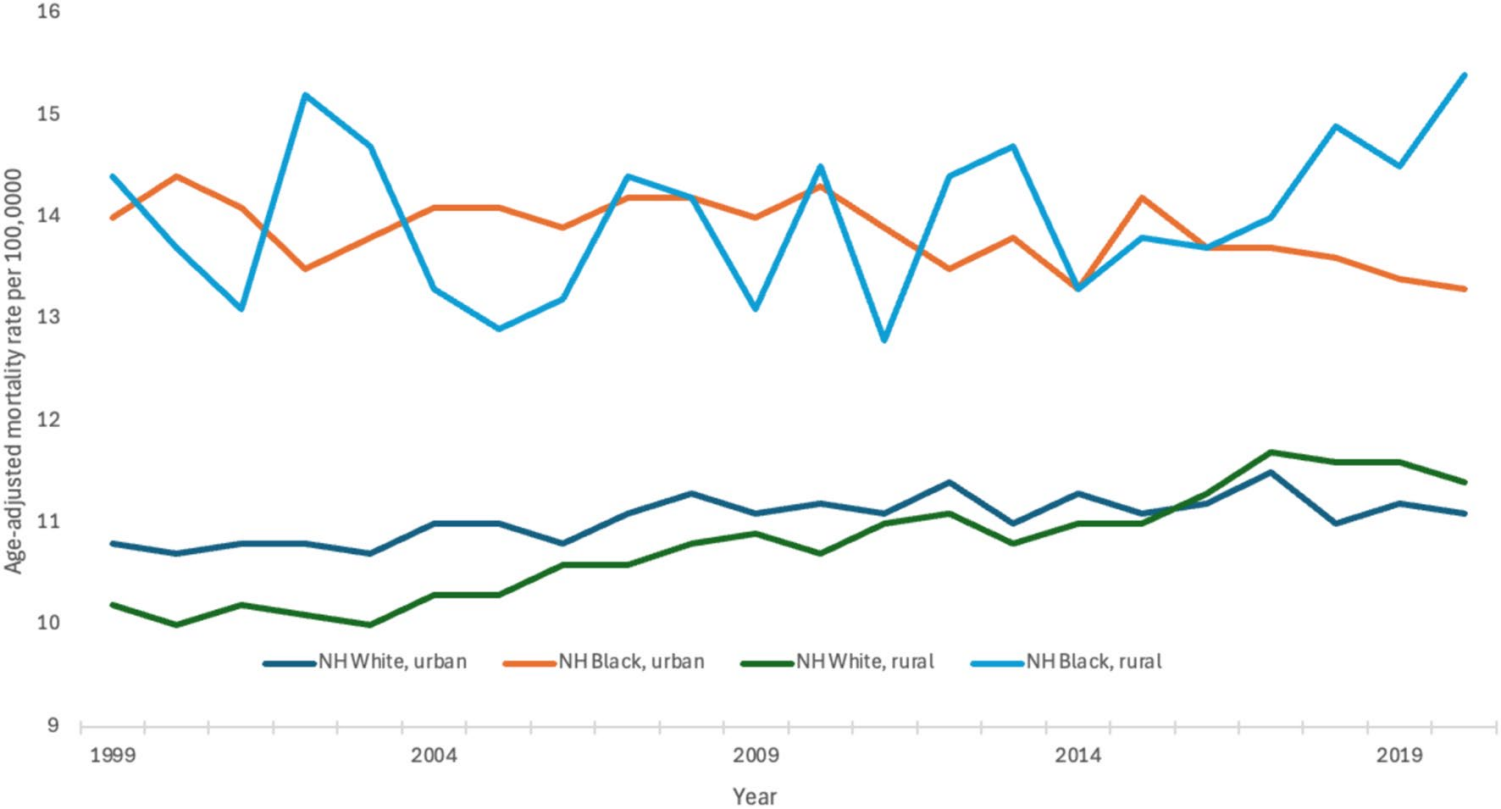
Trends in age‐adjusted, pancreatic cancer‐related mortality rates stratified by race and geographic density in the United States, 1999–2020. NH White, urban: 1999–2012 APC 0.4* (95% CI 0.2–1.6), 2012–2020 APC −0.1 (95% CI −1.5 to 0.2); NH White, rural: 1999–2020 APC 0.7* (95% CI 0.6–0.9); NH Black, urban: 1999–2020 APC −0.2* (95% CI −0.4 to −0.0); NH Black, rural: 1999–2020 APC 0.2 (95% CI −0.2 to 0.8)

For Non-Hispanic White individuals in urban populations, the AAMR rose from 10.8 in 1999 to 11.1 in 2020 with an APC of 0.2 (95% CI 0.1–0.4). Non-Hispanic White individuals in rural populations saw an increase in the AAMR from 10.2 in 1999 to 11.4 in 2020 with an APC of 0.7 (95% CI 0.6–0.9). Non-Hispanic Black individuals in urban populations experienced a decrease in the AAMR from 14.0 in 1999 to 13.3 in 2020 with an APC of -0.2 (95% CI −0.4 to 0.0). Non-Hispanic Black individuals in rural populations experienced an increase in the AAMR from 14.4 in 1999 to 15.4 in 2020; however, this trend was not statistically significant (Fig. 4).

**Fig. 5:**
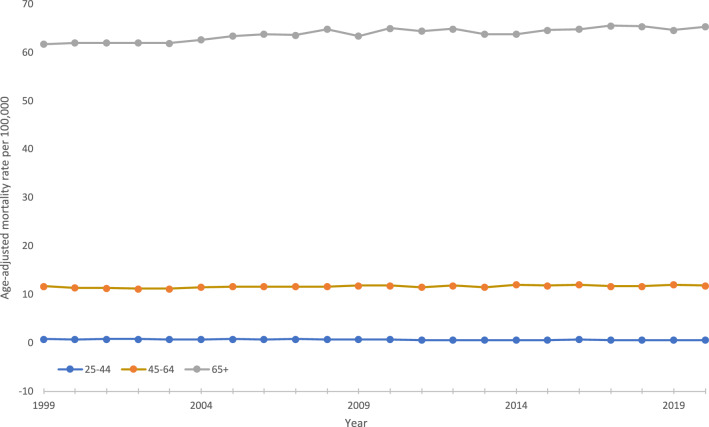
Trends in age‐adjusted, pancreatic cancer‐related mortality rates stratified by age in the United States, 1999–2020. 25–44 years: 1999–2020 APC −1.4* (95% CI −1.9 to −0.9); 45–64 years: 1999–2020 APC 0.2* (95% CI 0.1–0.3); 65+ years: 1999–2020 APC 0.3* (95% CI 0.2–0.3). *Indicates that the annual percentage change (APC) is significantly different from 0 at *α* = 0.05

During the study period, the average AAMR for adults aged 25–44 was 0.7, 11.7 for adults aged 45–64, and 63.9 for adults older than 65. The AAMR in those aged 25–44 decreased from 0.8 to 0.6 with an associated APC of -1.4 (95% CI −1.9 to −0.9). In adults aged 45–64, the AAMR slightly rose from 11.7 to 11.8 with an APC of 0.2 (95% CI 0.1–0.3), while in adults older than 65 the AAMR rose from 61.7 to 65.3 with an APC of 0.3 (95% CI 0.2–0.3) (Fig. 5).

**Fig. 6:**
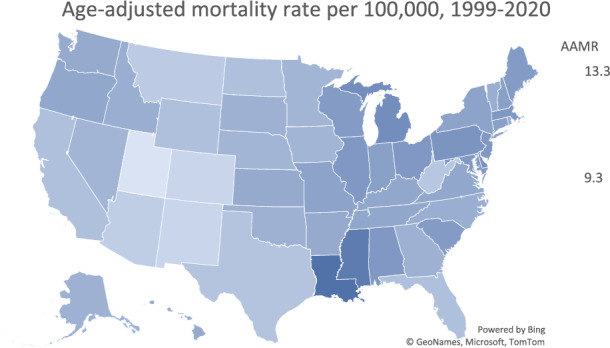
State‐level, age‐adjusted, pancreatic cancer‐related mortality rates in the United States from 1999 to 2020

Between 1999 and 2020, the Northeast experienced the highest pancreatic cancer related AAMR at 11.3, while the West experienced the lowest AAMR at 10.3. In the Northeast, the AAMR remained stable at 11.3 with an APC of 0.12 (95% CI 0.0–0.2). The Midwest experienced the most rapid increase in AAMR, from 10.6 in 1999 to 11.9 in 2020 with an APC of 0.51 (95% CI 0.4–0.6). The AAMR in the South rose from 10.7 in 1999 to 11.0 in 2020 with an APC of 0.24 (95% CI 0.1–0.3). In the West, the AAMR remained stable, rising slightly from 10.0 in 1999 to 10.3 in 2020 with an APC of 0.09 (95% CI −0.1 to 0.2). States and districts in the 90th percentile of mortality included Louisiana, District of Columbia, and Mississippi, while states in the 10th percentile of mortality included Utah, New Mexico, and Colorado (Fig. 6).

## Discussion

In this nationwide study of death certificates related to pancreatic cancer, we found several important insights using the CDC WONDER dataset to characterize pancreatic cancer mortality in the United States between 1999 and 2020. First, pancreatic cancer-related mortality in the United States has steadily risen at an average of 0.2% each year since 1999. Second, over the recent two decades, men have had a higher age-adjusted pancreatic cancer mortality rate compared to women (12.5 vs. 9.5 per 100,000). Third, Non-Hispanic Black individuals experienced the highest mortality of any race/ethnicity at 13.8. Fourth, the pancreatic cancer mortality decreased in young adults, while steadily rising in middle-aged and elderly adults. Fifth, the Northeast had the highest pancreatic cancer mortality between 1999 and 2020, while the Midwest experienced the most rapid rise during the same time frame.

Our results illustrate a significant increase in pancreatic cancer mortality in the United States since 1999, which may be driven by an increase in incidence, given that previous studies have shown an increase in incidence of 1.6% annually [14, 15]. An age and sex-specified time trend analysis of pancreatic cancer incidence between 2000 and 2018 found that the incidence of pancreatic cancer in all groups has increased since 2000 with an APC of 0.9%, with younger adults representing the most rapid increase [16]. However, older adults (> 55 years) make up most of the composition of pancreatic cancer cases in the United States, and incidence has risen with APCs of 0.92 in men and 0.62 in women [16]. In our study, the majority of pancreatic cancer mortality was observed in adults aged > 65 years – older adults accounted for 72% of total pancreatic cancer related deaths between 1999 and 2020 and had the highest average AAMR at 63.9. Thus, a driver for the rising AAMR seen in our study may largely be due to deaths in older adults. Interestingly, adults aged between 25 and 44 in our study experienced a slight decrease in mortality, from 0.8 to 0.6. This may coincidence with a slight increase in survival that has been reported in this population previously, possibly due to improvements in systemic therapies, advancements in imaging, and shifts in perioperative management [17].

Our study identified racial disparities present in pancreatic cancer mortality rates. These disparities may stem from disparities in incidence rates [18]. Between 2000 and 2018, Black females had an incidence rate of 14.41 compared to White females whose incidence rate was 11.11 [18]. This trend was mirrored in males as well where Black males had an incidence rate of 17.25 compared to White males whose incidence rate was 14.6 [18]. This disparity in pancreatic cancer incidence was similarly seen in mortality. This may be because Black individuals have an incidence rate of 12.8 per 100,000 of distant disease at diagnosis compared to White individuals with an incidence rate of 9.7 per 100,000 [14]. Reasons for this disparity in survival may be tied to racial disparities in the clinical trials processes. Between 2005 and 2020, White patients comprised 84.7% of clinical trial participants while Black patients only comprised 8.2% of participants [19]. Additional upstream factors leading to the racial disparities in pancreatic cancer-related mortality and incidence may also be explained by disparities in risk factors associated with pancreatic cancer. Black individuals not only have a higher mortality rate from pancreatic cancer, but they are also more likely to experience known risk factors such as obesity and smoking, which may help to explain potential complications associated with treatment of their cancer which subsequently led to the poorer outcomes seen in these groups [20].

Moreover, our data show disparities in mortality when comparing rural individuals with their urban and suburban counterparts. Pancreatic cancer mortality in the urban subgroup was found to slightly increase from 1999 to 2011 with an APC of 0.20. Then, in 2011, it fell with an APC of −0.22. In spite of this, our results show that the overall mortality increased from 1999 to 2020 in both the suburban and rural subgroups, with the rural subgroup having the highest APC of any subgroups at 0.6. Previous studies have shown that individuals residing in rural areas have the lowest rates of local or locoregional stage diagnosis, at 25% compared with 10% nationally [21]. They also found that rural individuals receiving surgery live farther from major tertiary care high-volume academic medical centers and be operated on at low volume hospitals which have been associated with worse healthcare outcomes compared to higher volume hospitals [22]. In our study, the rural subgroup experienced a notable spike in mortality where it rose at 1.9% annually between 2014 and 2017, which may be due to decreased access to newer systemic therapies and those used to treat metastatic disease [23]. Considering our results and existing literature, the need for reform and improved access to adequate care in rural communities remains an unmet need. Strategies including improving health literacy in rural populations and expanding screening access may be explored to improve outcomes for these communities [24, 25].

We assessed the intersection of rurality and race in the Non-Hispanic White and Non-Hispanic Black subgroups. We found that Non-Hispanic White individuals in both urban and rural populations saw an increase in mortality during the study period, with the rural subgroup experiencing a steeper rate of increase. Non-Hispanic Black individuals in urban environments saw a decrease in mortality, whereas those in rural populations saw a stable mortality trend. Our results are similar to those of a Ma and colleagues [26]. Interestingly, their study identified an increasing mortality trend in Non-Hispanic Black women in rural areas, compared with a stable trend in Non-Hispanic Black men in rural areas, which may be due to targeted interventions for smoking and obesity in this population [26]. Our study compared the most urban populations (metro populations > 1 million) with the most rural populations (nonmetro with populations < 50,000), whereas Ma and colleagues assessed by metro or nonmetro status, which may explain differences in APC seen when comparing our results.

Since 1999, a number of surgical and oncological advancements for pancreatic cancer treatment have been made [27]. One of the key surgical strategies is the evolution of enhanced recovery after surgery (ERAS) programs. ERAS programs aim to decrease the body’s stress response to surgery, which allows for a faster recovery [28]. These programs have been shown to be safe and feasible, while reducing complications and improving survival [28]. Additionally, a number of oncological advancements have been made since 1999. The first of these was the approval of erlotinib, an epidermal growth factor receptor (EGFR) inhibitor, in combination with gemcitabine for treatment of advanced pancreatic cancer [29]. Next was the phase III trial in 2013 that led to the FDA approval of nab-paclitaxel in 2014 [30]. Most recently, a phase III clinical trial assessing nanoliposomal irinotecan in combination with fluorouracil and leucovorin for treatment of metastatic pancreatic cancer that has progressed after gemcitabine-based chemotherapy demonstrated favorable results [31]. However, despite advancements in treatment, pancreatic cancer survival remains dismal and mortality continues to climb.

Our study is not without limitations. First, the CDC WONDER database utilizes data collected from death certificates to determine cause of death; thus, subclinical pancreatic cancer diagnosed at autopsy may have inflated mortality statistics [32]. However, we opted to use this database because it contains granular geographic information not contained in any other national database including the National Cancer Database. Second, the CDC WONDER database does not capture relevant information regarding clinical or pathologic disease characteristics, including anatomical site, histology, grade, or presence of distant metastases. Additionally, because the database reports the state in which death occurred, it is possible that deaths may be misclassified from the state that the individual resided in, which may impact geographic trends. Lastly, it is possible that the database may have misclassified cause of death on the death certificates reported. However, the CDC WONDER database is a comprehensive source of data that has been widely used in studies assessing cancer and other causes of mortality [6, 7, 33].

## Conclusion

Our study identifies a concerning increasing trend in pancreatic cancer mortality in the United States over the last twenty years which may be tied with increasing incidence rates during this time period. Further, we identified a number of demographic and regional disparities in pancreatic cancer mortality, including higher mortality in men, Non-Hispanic Black individuals, and in the geographic South. Lastly, we assessed the intersection of race and rurality, finding contrasting emerging trends between Non-Hispanic White and Black populations. Future research should consider focusing on mitigating drivers of mortality, including those that specifically affect vulnerable populations, such as financial barriers to care and clinical trial participation in underrepresented minority groups.

## Data Availability

The data that support the findings of this study are openly available at https://wonder.cdc.gov.
